# Metabolic syndrome, fatty liver, and artificial intelligence-based epicardial adipose tissue measures predict long-term risk of cardiac events: a prospective study

**DOI:** 10.1186/s12933-021-01220-x

**Published:** 2021-01-29

**Authors:** Andrew Lin, Nathan D. Wong, Aryabod Razipour, Priscilla A. McElhinney, Frederic Commandeur, Sebastien J. Cadet, Heidi Gransar, Xi Chen, Stephanie Cantu, Robert J. H. Miller, Nitesh Nerlekar, Dennis T. L. Wong, Piotr J. Slomka, Alan Rozanski, Balaji K. Tamarappoo, Daniel S. Berman, Damini Dey

**Affiliations:** 1 Biomedical Imaging Research Institute, Cedars-Sinai Medical Center, 116 N Robertson Boulevard, Los Angeles, CA 90048 USA; 2 Monash Cardiovascular Research Centre, MonashHeart, Clayton, Victoria Australia; 3 Heart Disease Prevention Program, Division of Cardiology, University of California at Irvine, Irvine, CA USA; 4 Department of Imaging and Medicine and the Smidt Heart Institute, Cedars-Sinai Medical Center, Los Angeles, CA USA; 5 Division of Cardiology, Mount Sinai St Lukes Hospital, New York, NY USA

**Keywords:** Metabolic syndrome, Non-alcoholic fatty liver disease, Epicardial adipose tissue, Artificial intelligence, Cardiovascular risk

## Abstract

**Background:**

We sought to evaluate the association of metabolic syndrome (MetS) and computed tomography (CT)-derived cardiometabolic biomarkers (non-alcoholic fatty liver disease [NAFLD] and epicardial adipose tissue [EAT] measures) with long-term risk of major adverse cardiovascular events (MACE) in asymptomatic individuals.

**Methods:**

This was a post-hoc analysis of the prospective EISNER (Early-Identification of Subclinical Atherosclerosis by Noninvasive Imaging Research) study of participants who underwent baseline coronary artery calcium (CAC) scoring CT and 14-year follow-up for MACE (myocardial infarction, late revascularization, or cardiac death). EAT volume (cm^3^) and attenuation (Hounsfield units [HU]) were quantified from CT using fully automated deep learning software (< 30 s per case). NAFLD was defined as liver-to-spleen attenuation ratio < 1.0 and/or average liver attenuation < 40 HU.

**Results:**

In the final population of 2068 participants (59% males, 56 ± 9 years), those with MetS (n = 280;13.5%) had a greater prevalence of NAFLD (26.0% vs. 9.9%), higher EAT volume (114.1 cm^3^ vs. 73.7 cm^3^), and lower EAT attenuation (−76.9 HU vs. −73.4 HU; all p < 0.001) compared to those without MetS. At 14 ± 3 years, MACE occurred in 223 (10.8%) participants. In multivariable Cox regression, MetS was associated with increased risk of MACE (HR 1.58 [95% CI 1.10–2.27], p = 0.01) independently of CAC score; however, not after adjustment for EAT measures (p = 0.27). In a separate Cox analysis, NAFLD predicted MACE (HR 1.78 [95% CI 1.21–2.61], p = 0.003) independently of MetS, CAC score, and EAT measures. Addition of EAT volume to current risk assessment tools resulted in significant net reclassification improvement for MACE (22% over ASCVD risk score; 17% over ASCVD risk score plus CAC score).

**Conclusions:**

MetS, NAFLD, and artificial intelligence-based EAT measures predict long-term MACE risk in asymptomatic individuals. Imaging biomarkers of cardiometabolic disease have the potential for integration into routine reporting of CAC scoring CT to enhance cardiovascular risk stratification.

*Trial registration* NCT00927693.

## Introduction

The metabolic syndrome (MetS) is a cluster of atherosclerotic cardiovascular disease (ASCVD) risk factors centered on obesity, hypertension, hyperglycemia and atherogenic dyslipidemia, and carries a proinflammatory state [1]. Currently, MetS affects close to one-third of adults in the United States [2] and is associated with significant cardiovascular morbidity and mortality [3]. There has been much research interest into visceral fat accumulation associated with the MetS, with non-alcoholic fatty liver disease (NAFLD) and epicardial adipose tissue (EAT) emerging as clinical markers of cardiometablic risk [4–7]. Noncontrast cardiac computed tomography (CT), routinely used for coronary artery calcium (CAC) scoring, enables the noninvasive diagnosis of NAFLD and quantification of EAT. The presence of liver fat is detected as a decreased liver CT attenuation [8], while EAT volume and attenuation can be measured using semi- or fully-automated software applications [9, 10]. While these CT-derived metrics have been shown to individually associate with incident coronary artery disease (CAD) and cardiovascular events [11–14], no studies have examined the prognostic effect of Mets, NAFLD and EAT simultaneously in asymptomatic individuals. The prospective EISNER (Early-Identification of Subclinical Atherosclerosis by Noninvasive Imaging Research) registry [12, 15] comprised a large community-based cohort with no CAD who underwent CAC scoring CT and 14-year follow-up for cardiac events. In this post-hoc analysis, we sought to determine the long-term prognostic value of MetS and CT biomarkers of cardiometabolic disease (liver fat and artificial intelligence (AI)-based EAT measures) in EISNER participants.

## Methods

### Study population

We studied 2651 participants from the prospective EISNER registry [12, 15] at Cedars-Sinai Medical Center (CSMC). Inclusion criteria for the EISNER registry were: age 45–80 years and intermediate risk of CAD based on age (> 55 years in men, > 65 years in women) or the presence of at least one CAD risk factor in younger individuals (age 45–54 years in men or 55–64 years in women). Exclusion criteria were: history of cardiac or cerebrovascular disease or chest pain, prior CAC scanning or invasive coronary angiography, or significant medical co-morbidity. All participants underwent baseline CAC scoring CT and clinical evaluation.

### Prognostic follow-up

Participants were prospectively followed up during a mean of 14 ± 3 years for major adverse cardiovascular events (MACE), defined as myocardial infarction (MI), late revascularization (occurring > 180 days after the CT), or cardiac death. Complete outcomes data were obtained in 2068 (78%) individuals. Follow-up was via clinical visits, detailed questionnaires sent by mail, or telephone contact. Reported event information was verified by the National Death Index query and by comprehensive review of electronic medical, hospital, and death records by 2 independent cardiologists blinded to clinical data. The research was approved by the CSMC Institutional Review Board and all participants provided written informed consent.

### Ascertainment of risk factors at baseline

Detailed information was obtained from all participants on co-morbidities, smoking history, alcohol consumption, and medications. Measurements were obtained for body mass index (BMI); blood pressure; fasting total, high-density lipoprotein (HDL) and low-density lipoprotein (LDL) cholesterol; triglycerides; and serum glucose. The Pooled Cohort Equation [16] was used to calculate the 10-year risk of ASCVD.

### Definition of the metabolic syndrome

MetS was defined by the International Diabetes Federation (IDF) worldwide definition [17]: body mass index (BMI) ≥ 30 kg/m^2^ (in which case, central obesity can be assumed and waist circumference need not be measured) plus any two of the following factors: (1) raised triglycerides (≥ 150 mg/dL) or specific treatment for this lipid abnormality; (2) reduced HDL cholesterol (< 40 mg/dL in males; < 50 mg/dL in females) or specific treatment; (3) raised blood pressure (systolic ≥ 130 mmHg or diastolic ≥ 85 mmHg) or treatment for previously diagnosed hypertension; and (4) elevated fasting plasma glucose (≥ 100 mg/dL) or previously diagnosed type 2 diabetes. As waist circumference data were not uniformly available for the EISNER cohort, we used modified IDF criteria wherein BMI ≥ 30 kg/m^2^ assumed central obesity [17].

### Image acquisition

Noncontrast cardiac CT scans were performed on an Electron Beam CT scanner (e-Speed, GE Healthcare, Milwaukee, WI, USA) or 4-slice CT scanner (Somatom Volumezoom, Siemens Medical Solutions, Erlangen, Germany). During a single breath hold, 40-50 electrocardiogram-gated slices were acquired from the carina to below the apex of the heart; tube voltage was 120 kVp and reconstruction slice thickness was either 2.0, 2.5, or 3.0 mm.

### CAC measurement

Scans were analyzed by cardiologists using commercially available semi-automated CAC scoring software (ScImage Inc., Los Altos, CA, USA). The per-patient CAC score was measured according to the Agatston method [18]. CAC volume (cm^3^) per-vessel was computed by the software as the total volume of calcified lesions along the vessel. Per-vessel area scores (cm^2^) were calculated by dividing the CAC volume by the appropriate slice thickness. Calcium density score was calculated per-vessel by dividing the Agatston score by the area score; the density score ranged from 1 to 4 and reflected the average plaque density [19].

### Deep learning-based EAT quantification

**Fig. 1:**
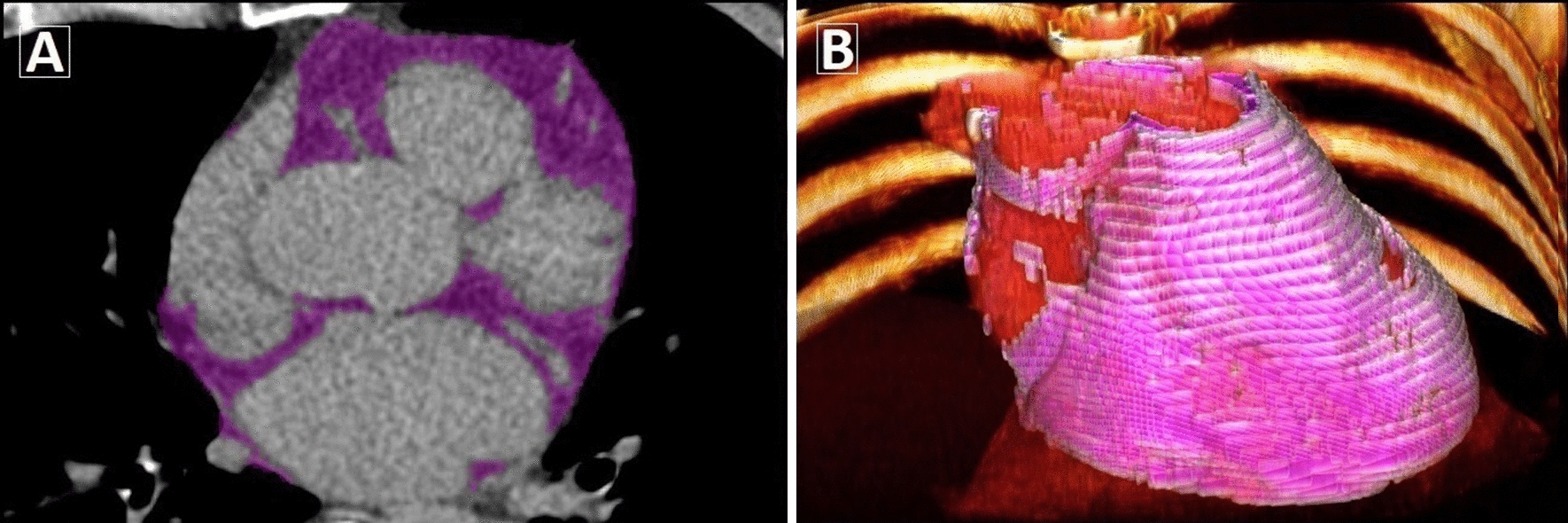
Artificial intelligence-based EAT quantification. Case example of fully automated EAT segmentation (purple) from noncontrast CT using deep learning software (**a**); with 3D volume rendering of EAT shown in pink (**b**)

EAT was defined as all adipose tissue enclosed by the visceral pericardium. EAT volume and attenuation were quantified using a fully automated deep learning (DL) algorithm [10] incorporated into research software (QFAT v2.0, CSMC, Los Angeles, CA, USA) (Fig. 1a and b). This DL algorithm was validated and tested in a large multicenter study [10] and has also demonstrated predictive value for MACE in individuals from the EISNER registry [14].

For regional analysis of EAT measures, eight EAT segments based from the center of the heart in the axial view were automatically generated by DL. EAT segments were assigned to the best suitable coronary artery (left anterior descending artery [LAD], left circumflex artery [LCx], right coronary artery [RCA]) according to myocardial vascular territories [20]. Regional EAT volume corresponding to each major artery was calculated from the vessel-assigned EAT segments; EAT attenuation was the mean attenuation of these regions (Additional file 1: Figure S1). The processing time for DL-based quantification of EAT was approximately 25 s per case.

### Liver fat measurement

For the CT-based assessment of NAFLD, we excluded 66 participants: 41 with heavy alcohol intake (> 21 standard drinks per week for men and > 14 drinks per week for women) [21], 22 on oral corticosteroids, and 3 on oral amiodarone. In the remaining 2002 participants, two independent readers blinded to clinical data performed manual measurements of liver fat on noncontrast CT images using QFAT v2.0. Hepatic and splenic HU attenuation values were quantified using regions of interest (ROI) > 100 mm^2^ in area. In the same axial slice, two ROI were placed in the right liver lobe anteroposteriorly, one ROI in the left liver lobe, and one ROI in the spleen (Additional file 1: Figure S2). Liver-to-spleen ratio was calculated by taking the mean HU measurement of both right liver lobe ROIs and dividing it by the splenic HU. Average liver attenuation was the mean HU of right and left lobe ROIs. NAFLD was defined as liver-to-spleen ratio < 1.0 and/or average liver attenuation < 40 HU [8, 22].

### Serum biomarkers

Serum samples were collected at the time of CT, centrifuged and stored in a −80 ℃ freezer biobank until assayed. In a subset of 1069 participants, biomarkers of inflammation (high-sensitivity C-reactive protein [hs-CRP], interleukin 6 [IL-6], myeloperoxidase [MPO]), thrombosis (plasminogen activator inhibitor 1 [PAI-1], D-dimer), and atherosclerosis (endothelial cell-selective adhesion molecule [ESAM], lymphotoxin β receptor [LTBR]), as well as the vasoprotective adiponectin, were measured by an independent and blinded laboratory (Alere™ Inc., San Diego, CA, USA) using sandwich enzyme-linked immunosorbent assays.

### Statistical analysis

Continuous variables are reported as mean ± standard deviation or median (interquartile range). Distributions of CAC score and EAT volume were not normally distributed and hence normalized with logarithmic adjustment; base-2 logarithmic transformation was used as this represented doubling of the variable. Pearson or Spearman’s rank correlations were used to assess correlations between continuous variables. Multivariable linear regression was used to evaluate the relationship between regional EAT attenuation and per-vessel calcium density score, with adjustment for log-transformed per-vessel CAC volume. Binary logistic regression was used to evaluate the association of EAT measures with presence of MetS or NAFLD.

Multivariable Cox regression analysis with backward stepwise selection was used to determine the association of MetS with risk of MACE, adjusted for age, male sex, smoking, LDL cholesterol level, statin use, and antihypertensive treatment. Given that MetS is a predictor of type 2 diabetes [23], we examined risks in individuals defined with MetS (but without diabetes) and diabetics as separate categories, compared to an “optimal” risk reference group with neither condition (Model 1). We then adjusted for CAC score (Model 2), CAC score plus EAT volume (Model 3), and CAC score plus EAT attenuation (Model 4). The independent effects of NAFLD and liver attenuation on MACE were assessed using backward stepwise Cox regression, with adjustment for MetS, CAC score, and EAT volume or attenuation.

The continuous net reclassification index (NRI) [24] was used to measure the incremental prognostic value of adding EAT volume or attenuation to current risk assessment tools (ASCVD risk score alone or in combination with CAC score). Individuals were classified into ‘high’ or ‘low’ EAT volumes based on a cutoff of ≥ 113 cm^3^ determined by the maximum Youden’s index (sum of sensitivity + specificity) on receiver operating characteristic (ROC) curve analysis for MACE. Kaplan-Meier survival curves were calculated for individuals stratified by high versus low EAT volume and presence or absence of NAFLD; the log-rank test was used to compare survival distributions. Analyses were performed using Stata/IC 15.1 (StataCorp LP, College Station, TX, USA), with SAS 9.4 (SAS Institute, Cary, NC, USA) used for NRI computation. A 2-sided p-value of < 0.05 indicated statistical significance.

## Results

### Baseline characteristics

The final study population consisted of 2068 participants with mean age 55.6 ± 9.1 years and 59% males. Based on IDF criteria, 280 (13.5%) participants had MetS, of whom 234 (83.6%) had no diabetes. Characteristics of individuals with and without MetS are summarized in Table 1. At mean follow-up of 14 ± 3 years, 223 (10.8%) participants suffered MACE. Of these, 42 (18.8%) had MI, 145 (65.0%) underwent late revascularization, and 36 (16.1%) experienced cardiac death.

**Table 1:** Baseline characteristics of the study population

	Total participants	MetS	No MetS	*P* value
	(n = 2068)	(n = 280)	(n = 1788)	
Demographics				
Age, years	55.6 ± 9.1	56.4 ± 8.4	55.5 ± 9.2	0.10
Male gender	1220 (59.0)	166 (59.3)	1060 (59.3)	1.00
BMI, kg/m^2^	26.6 ± 4.9	34.9 ± 5.2	25.3 ± 3.3	< 0.001
Systolic blood pressure, mmHg	128.8 ± 18.4	137.6 ± 17.5	127.4 ± 18.2	< 0.001
Diastolic blood pressure, mmHg	77.1 ± 11.8	81.9 ± 12.3	76.3 ± 11.6	< 0.001
Dyslipidemia	1439 (69.6)	212 (75.7)	1227 (68.6)	0.02
Hypertension	834 (40.3)	186 (66.4)	648 (36.2)	< 0.001
Diabetes mellitus	119 (5.8)	46 (16.4)	73 (4.1)	< 0.001
Family history of CAD	623 (30.1)	79 (28.2)	544 (30.4)	0.48
Current smoker	48 (2.3)	8 (2.9)	40 (2.2)	0.19
Past smoker	81 (3.9)	12 (4.3)	69 (3.9)	0.26
ASCVD risk, %	5.2 (2.6–10.1)	8.1 (4.8–15.2)	4.8 (2.3–9.4)	< 0.001
Medications				
Aspirin	244 (11.8)	51 (18.4)	193 (10.8)	0.005
Statin	449 (21.7)	93 (33.2)	356 (19.9)	< 0.001
ACE-inhibitor/ARB	175 (8.5)	51 (18.2)	124 (6.9)	< 0.001
Beta blocker	145 (7.0)	30 (10.8)	115 (6.4)	0.04
Diuretic	157 (7.6)	40 (14.3)	117 (6.5)	0.001
Quantitative CT measures				
CAC score	0 (0–56.6)	5.6 (0–92.3)	0 (0–50.8)	0.001
CAC score category				
0	1084 (52.4)	123 (43.9)	961 (53.7)	0.002
1–100	590 (28.5)	90 (32.1)	500 (28.0)	0.16
101–400	241 (11.7)	43 (15.4)	198 (11.1)	0.05
> 400	153 (7.4)	24 (8.6)	129 (7.2)	0.39
EAT volume, cm^3^	78.3 (55.7–106.0)	114.1 (90.7–147.8)	73.7 (53.7–98.7)	< 0.001
EAT attenuation, HU	−73.8 ± 4.8	−76.9 ± 4.6	−73.4 ± 4.6	< 0.001
Liver attenuation, HU^a^	64.5±11.6	54.8±12.6	65.8 ± 10.3	< 0.001
NAFLD^a^	242/1962 (12.3)	71/273 (26.0)	171/1689 (10.1)	< 0.001
Laboratory values				
Total cholesterol, mg/dL	210.9 ± 40.1	210.5 ± 43.3	211.0 ± 39.6	0.85
LDL cholesterol, mg/dL	131.3 ± 37.2	130.5 ± 37.6	131.4 ± 37.1	0.71
HDL cholesterol, mg/dL	55.2 ± 17.1	44.7 ± 13.4	56.8 ± 17.1	< 0.001
Triglycerides, mg/dL	105.0 (74.0–152.0)	158.5 (114.5–213.0)	99.0 (70.0–140.0)	< 0.001
Fasting glucose, mg/dL	96.1 ± 16.9	102.8 ± 25.5	95.0 ± 14.8	< 0.001

Values are expressed as n (%), mean ± standard deviation, or median (interquartile range, 25th–75th)

^a^Data available in 1962 participants

ACE, angiotensin converting enzyme; ARB, angiotensin receptor blocker; ASCVD, atherosclerotic cardiovascular disease; BMI, body mass index; CAC, coronary artery calcium; CAD, coronary artery disease; CT, computed tomography; EAT, epicardial adipose tissue; HDL, high-density lipoprotein; HU, Hounsfield units; LDL, low-density lipoprotein; MetS, metabolic syndrome; NAFLD, non-alcoholic fatty liver disease

### Noncontrast CT measures

Median EAT volume was 78.3 (55.7–106.0) cm^3^ and mean EAT attenuation was 64.5 ± 11.6 HU. CAC was present in 984 (48%) individuals, who had a median CAC score of 63.8 (18.2–208.9). There was an inverse correlation between EAT volume and attenuation (r = −0.80, p < 0.001).

Attenuation measurements of both right and left liver lobes were available in 1962 participants, and splenic attenuation was available in 1395 participants. The prevalence of NAFLD was 12.3% (242/1962), with liver-to-spleen ratio < 1.0 in 11.4% (223/1962) and liver attenuation < 40 HU in 6.6% (129/1962). The median CAC score was higher in individuals with versus without NAFLD (7.5 [0–94.3] vs. 0 [0–52.1], p = 0.009).

### MetS and EAT measures as predictors of MACE

In multivariable Cox regression analysis, risk of MACE was increased in individuals with MetS and no diabetes (Table 2, Model 1), even after adjustment for CAC score (HR for MetS: 1.58 [95% CI 1.10-2.27], p = 0.01) (Table 2, Model 2). Further adjustment for EAT measures resulted in only CAC score and EAT volume/attenuation being significantly associated with MACE risk; MetS no longer had an independent prognostic effect (p = 0.27) (Table 2, Models 3 and 4).

**Table 2:** Association of MetS, CAC score, and EAT measures with MACE risk in multivariable Cox regression

	HR	95% CI	*P* value
Model 1			
MetS/diabetes status			
No MetS or diabetes	1.00 (Reference)		
MetS (no diabetes)	1.62	1.13–2.34	0.01
Diabetes	1.78	1.11–2.84	0.02
Age, years	1.07	1.05–1.08	< 0.001
Male sex	1.83	1.37–2.44	< 0.001
LDL cholesterol, mmol/L	1.02	1.01–1.03	0.01
Model 2: Model 1 + CAC score			
CAC score^a^	1.28	1.22–1.33	< 0.001
Mets/diabetes status			
No MetS or diabetes	1.00 (Reference)		
MetS (no diabetes)	1.58	1.10–2.27	0.01
Diabetes	1.44	0.90–2.30	0.13
Model 3: Model 2 + EAT volume^b^			
EAT volume, cm^3a^	1.52	1.23–1.89	< 0.001
CAC score	1.28	1.23–1.33	< 0.001
Model 4: Model 2 + EAT attenuation^b^			
EAT attenuation, HU	0.95	0.93–0.98	< 0.001
CAC score	1.27	1.21–1.32	< 0.001

Final models based on backward stepwise selection of variables at a Wald p-value of 0.05

Co-variates entered in Model 1: age, male sex, current smoker, past smoker, LDL cholesterol, statin use, anti-hypertensive treatment

^a^Hazard ratios are per 2-fold increase/doubling of EAT volume (cm^3^) and CAC score

^b^MetS/diabetes status categories were not significantly associated with MACE risk

CAC, coronary artery calcium; CAD, coronary artery disease; EAT, epicardial adipose tissue; LDL, low-density lipoprotein cholesterol; MACE, major adverse cardiovascular events; MetS, metabolic syndrome

Adding EAT volume to current risk assessment tools (ASCVD risk score alone or in combination with CAC score) resulted in substantial net reclassification improvement for MACE, with significant reclassification of events and non-events among participants. EAT attenuation also demonstrated incremental prognostic value beyond traditional risk assessment, driven primarily by reclassification of non-events (Table 3).

**Table 3:** Improvement in MACE risk reclassification using EAT measures beyond current risk assessment tools

	NRI	95% CI	*P* value	% of events correctly reclassified	Event *P* value	% of non-events correctly reclassified	Non-event *P* value
ASCVD risk score + EAT volume	0.218	0.079-0.357	0.002	14	0.04	8	< 0.001
ASCVD + CAC score + EAT volume	0.171	0.032-0.310	0.001	11	0.01	6	0.009
ASCVD risk score + EAT attenuation	0.167	0.038-0.31	0.01	5	0.08	12	< 0.001
ASCVD + CAC score + EAT attenuation	0.126	0.014-0.266	0.02	4	0.09	9	< 0.001

ASCVD, atherosclerotic cardiovascular disease; CAC, coronary artery calcium; EAT, epicardial adipose tissue; NRI, net reclassification index

### NAFLD as a predictor of MACE

In multivariable Cox analysis adjusted for MetS, CAC score, and EAT volume or attenuation, CT-defined NAFLD was independently predictive of MACE (Table 4, Models 1 and 2).

**Table 4:** Association of NAFLD with MACE risk in multivariable Cox regression

	HR	95% CI	*P* value
Model 1: adjusted for CAC score + EAT volume			
NAFLD	1.78	1.21–2.61	0.003
EAT volume, cm^3^^a^	1.48	1.18–1.86	0.001
CAC score^a^	1.28	1.23–1.33	< 0.001
Model 2: adjusted for CAC score + EAT attenuation			
NAFLD	1.80	1.23–2.65	0.003
EAT attenuation, HU	0.96	0.93–0.98	0.002
CAC score	1.28	1.23–1.34	< 0.001

Final models based on backward stepwise selection of variables at a Wald p-value of 0.05

Co-variates entered in both models: age, male sex, MetS/diabetes status (no Mets or diabetes; MetS without diabetes; diabetes), LDL cholesterol, current smoker, past smoker, statin use, antihypertensive treatment

^a^Hazard ratios are per 2-fold increase/doubling of EAT volume (cm^3^) and CAC score

CAC, coronary artery calcium; CT, computed tomography; EAT, epicardial adipose tissue; HU, Hounsfield units; MACE, major adverse cardiovascular events; MetS, metabolic syndrome; NAFLD, non-alcoholic fatty liver disease

### Kaplan-Meier survival analysis

**Fig. 2:**
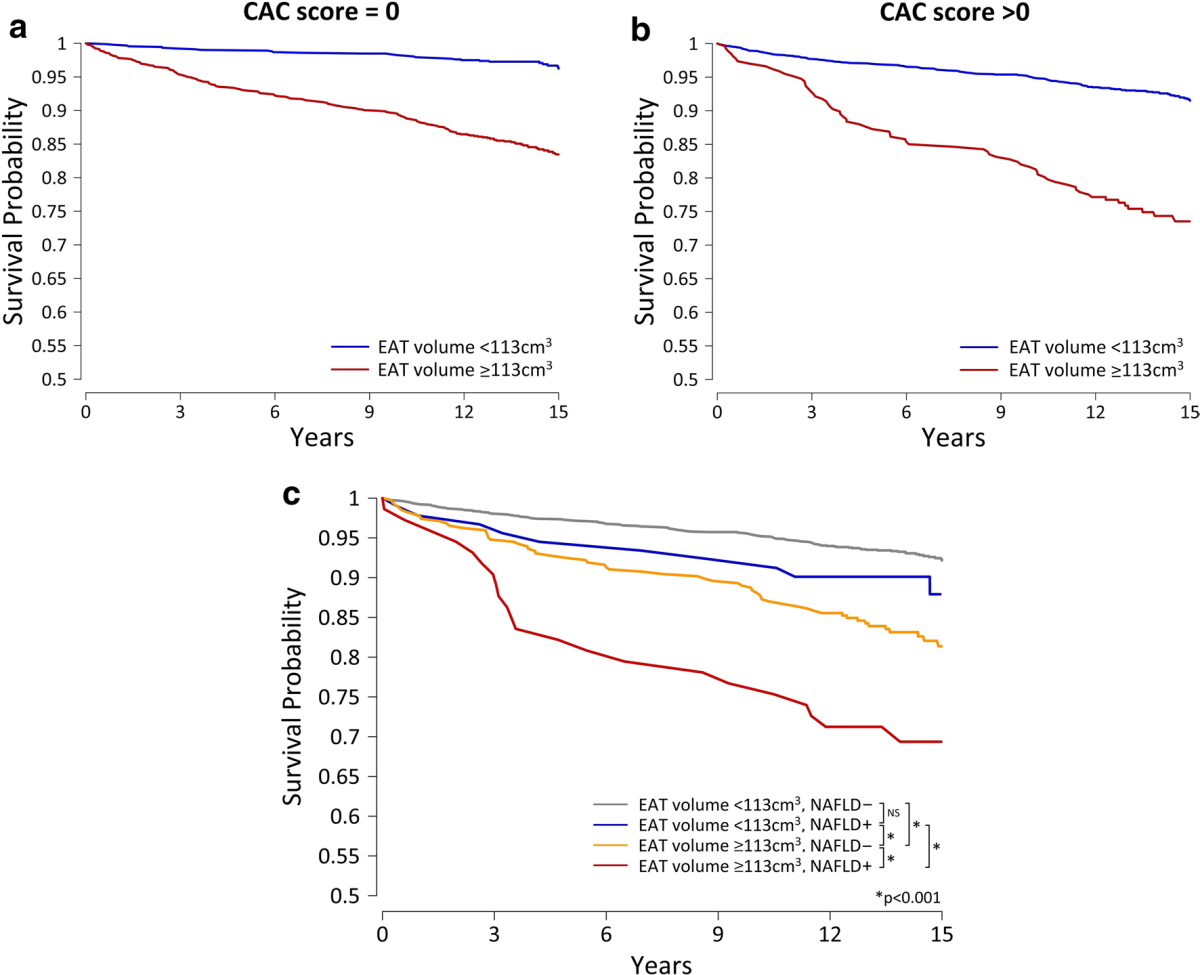
Kaplan-Meier curves of MACE by EAT volume and NAFLD. urvival curves for participants stratified by high (≥ 113 cm^3^) versus low (< 113 cm^3^) EAT volume in the absence (**a**) or presence (**b**) of CAC (both log-rank p< 0.001). The cumulative probability of survival was worst in individuals with a CAC score > 0 and high EAT volume. **c** Kaplan-Meier curves for participants stratified by high versus low EAT volume and presence of absence of NAFLD show survival probability to be worst in individuals with a high EAT volume and NAFLD (log-rank p-values displayed). CAC, coronary artery calcium; EAT, epicardial adipose tissue; MACE, major adverse cardiovascular events; NAFLD, non-alcoholic fatty liver disease; NS, non-significant

Individuals with a high EAT volume (≥ 113 cm^3^) had significantly worse MACE-free survival than those with a low EAT volume (< 113 cm^3^), both in the absence (Fig. 2a) and presence (Fig. 2b) of CAC (both p < 0.001). The cumulative probability of survival was worst in individuals with a CAC score > 0 and high EAT volume. Kaplan-Meier curves stratified by EAT volume and NAFLD are shown in Fig. 2c, with MACE-free survival probability being worst in individuals with both a high EAT volume and NAFLD.

### Association of EAT measures with MetS and NAFLD

**Fig. 3:**
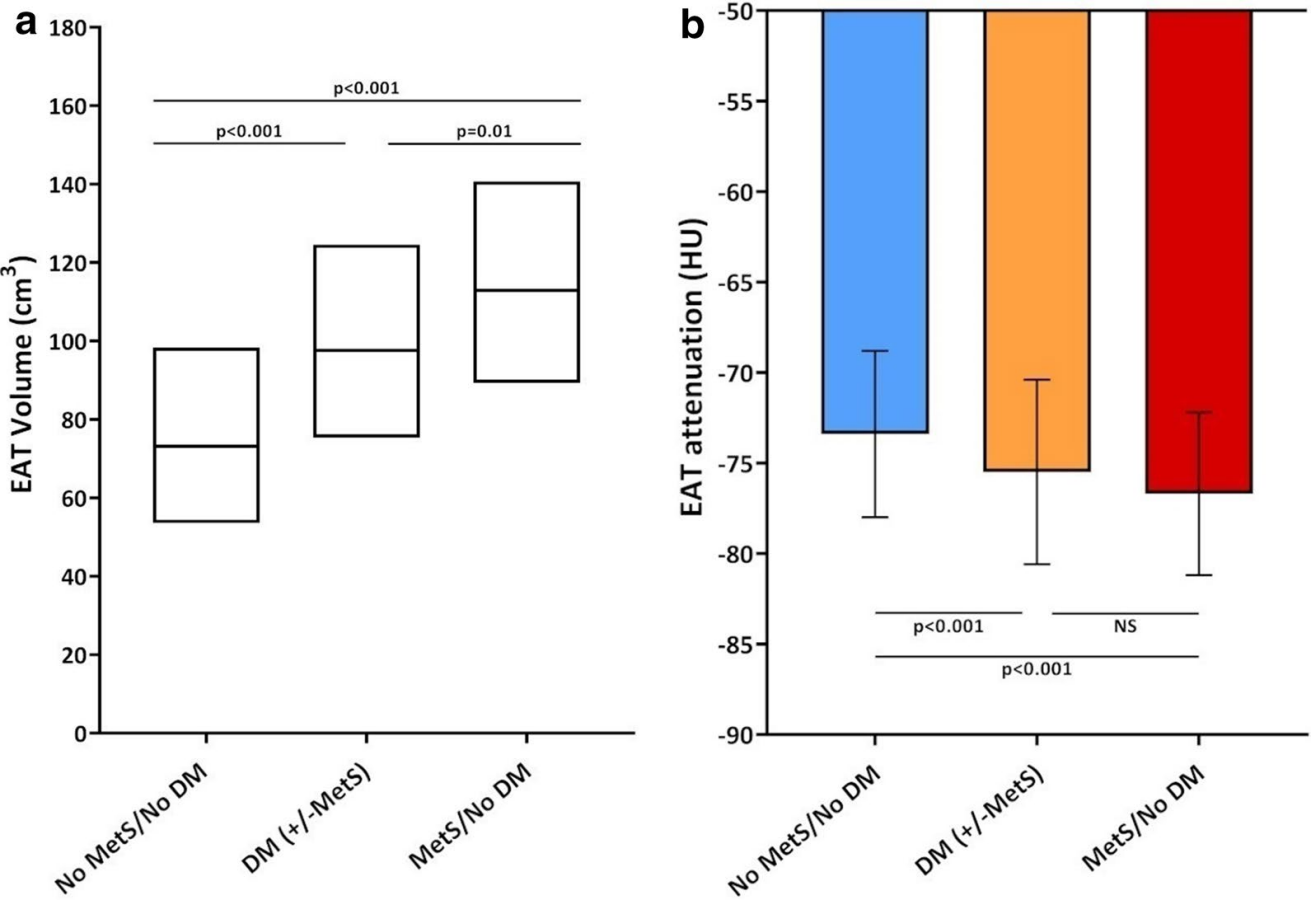
Relationship of EAT volume and attenuation with MetS and/or diabetes. In participants with: (1) neither MetS nor diabetes mellitus (DM), (2) DM (with or without MetS), and (3) MetS without DM, EAT volume (**c**) as shown in box plots was 73.2 [53.6–98.3] vs. 97.6 [75.4–124.6] vs. 112.9 [89.3–140.7] cm^3^, respectively (trend p < 0.001). EAT attenuation (**d**) was −73.4 ± 4.6 vs. −75.5 ± 5.1 vs. −76.7 ± 4.5 HU, respectively (trend p < 0.001). CT computed tomography; EAT, epicardial adipose tissue; HU, Hounsfield units; MetS, metabolic syndrome

AT volume and attenuation correlated with each individual component of the MetS **(**Additional file 1: Table S1). Multivariable logistic regression analysis showed EAT volume and attenuation to independently associate with the presence of MetS (Additional file 1: Table S2) and NAFLD (Additional file 1: Table S3). Subgroup analyses of participants with: (1) neither MetS nor diabetes (n = 1715), (2) diabetes (n = 119), and (3) MetS without diabetes (n = 234) revealed significant differences in EAT volume (73.2 vs. 97.6 vs. 112.9, trend p < 0.001) and attenuation (−73.4 ± 4.6 vs. −75.5±5.1 vs. −76.7±4.5 HU, trend p < 0.001) (Fig. 3a and b).

### Association of regional EAT measures with CAC characteristics

Regional EAT measures and per-vessel CAC characteristics are shown in (Additional file 1: Table S4). In bivariate analysis, EAT volume in all three vascular territories correlated with the CAC volume in corresponding arteries (LAD: r = 0.18; LCx: r = 0.17; RCA: r = 0.15, all p < 0.001). In linear regression analysis adjusted for per-vessel calcium volume, EAT attenuation in the LAD and RCA territories was associated with the per-vessel calcium density scores (standardized β of 0.179 and 0.210, respectively; both p < 0.001). EAT attenuation in the LCx territory was not associated with the LCx calcium density score (p = 0.38).

### MetS, NAFLD, and serum biomarker levels

Serums levels of inflammatory (hs-CRP, IL-6, MPO), thrombogenic (PAI-1, D-dimer), and novel atherosclerotic (ESAM, LTBR) biomarkers were higher in individuals with MetS compared to individuals without MetS. Conversely, levels of adiponectin were lower in the presence versus absence of the MetS. Similar results were observed for individuals with and without NAFLD (Additional file 1: Table S5).

## Discussion

In this study of asymptomatic individuals undergoing CAC scoring CT, the primary findings are: (1) AI-based quantification of EAT volume and attenuation significantly improve MACE risk reclassification over and above current risk assessment tools; (2) MetS is associated with increased risk of MACE at 14 years; however, not following adjustment for CT defined NAFLD or EAT measures; (3) NAFLD is a strong, independent long-term predictor of MACE.

### AI-Based EAT quantification

Earlier cohort studies showing noncontrast CT-derived EAT volume to associate with MetS and cardiac events used manual or semi-automated methods for EAT quantification [9, 12, 25, 26]. We recently reported that fully automated measurements of EAT volume and attenuation by DL software associate with MACE risk in asymptomatic individuals [14]. The present analysis extends these findings by demonstrating that AI-based EAT measures substantially improve risk reclassification for MACE over and above current risk prediction tools. We also examined the prognostic effect of EAT volume in asymptomatic participants with a CAC score of 0, given the existing evidence that such individuals have a very low long-term rate of cardiac events—the “power of zero” [27]. In this cohort, we demonstrated a high EAT volume to associate with significantly worse MACE-free survival compared to a low EAT volume. These findings may be partly explained by the effects of EAT on coronary plaque that are undetectable by CAC score screening. EAT volume associates more strongly with the presence of noncalcified plaque over calcified plaque on coronary CT angiography (CCTA) [28], and EAT volume and attenuation independently predict the presence of CCTA-derived high-risk plaque [29]. Finally, we showed our AI-based EAT metrics to be strong and independent correlates of the MetS and NAFLD. In subgroup analyses, EAT volume was greater in individuals with MetS or diabetes compared to individuals with neither condition, corroborating the results of previous studies [9, 30]. Such automated EAT measurements are rapid (< 30 s per case) and have the potential for integration into routine reporting of CAC scoring CT, providing real-time information on cardiometabolic risk.

### MetS and coronary atherosclerosis

In this study, participants with MetS had a higher median CAC score than those without MetS, lending support to the hypothesis that atherosclerosis is a pathophysiologic link between MetS and clinical events at a non-invasive imaging level. We showed MetS to confer an increased MACE risk independently of CAC score, suggesting that MetS also influences the non-calcified components of coronary plaques. Certainly, intracoronary imaging has demonstrated that plaques in MetS have a greater lipid burden compared with plaques in controls [31]. On CCTA, individuals with MetS are more likely than those without MetS to have non-calcified and high-risk plaques [32, 33]. Beyond coronary atherosclerosis, MetS also associates with myocardial steatosis and subclinical myocardial dysfunction [34], which portend worse cardiac outcomes. Understanding the mechanisms by which MetS modulates cardiovascular risk is particularly important, given the recent evidence that targeted lifestyle and dietary interventions can effectively treat the MetS [35, 36].

### Association of NAFLD with cardiac events

NAFLD, encompassing a continuum of liver diseases ranging from steatosis to cirrhosis, is regarded as both a cause and consequence of MetS [4]. The prevalence of both MetS and NAFLD increases with obesity, and there is a bidirectional association between NAFLD and individual components of the MetS [4]. The increased risk for atherosclerosis progression [37] and cardiac events conferred by NAFLD is well established [38], however only one prior report has examined the prognostic effect of CT defined liver fat. In a post-hoc analysis of the MESA study (Multi-Ethnic Study of Atherosclerosis), Zeb et al. [11] showed NAFLD diagnosed on noncontrast CT to associate with increased risk of non-fatal ischemic events and all-cause mortality at a median of 7.6 years, after adjustment for clinical risk factors. The present study demonstrates NAFLD detected on CT to be a strong predictor of MACE at 14-year follow-up, independently of MetS, CAC score, and EAT measures. Consistent with previous reports [39], we observed a higher burden of CAC in individuals with versus without NAFLD. Beyond CAC, studies have demonstrated an association of NAFLD with obstructive CAD [40] and high-risk plaques [41]. We found higher serum levels of ESAM and LTBR in the presence versus absence of MetS or NAFLD. These novel atherosclerosis biomarkers have been shown to predict prevalent CAC [42, 43] and incident ASCVD [44] in asymptomatic patients, and could potentially play a distinct atherogenic role in those with increased cardiometabolic risk.

### Adipose tissue inflammation

Chronic, low-grade inflammation may be an important pathophysiologic link between MetS, NAFLD, EAT, and cardiac events in our study. In keeping with prior studies [45, 46], we found higher serum levels of hs-CRP in individuals with MetS or NAFLD compared to those without. Obesity induces adverse remodeling of visceral adipose tissue, leading to expression of a proinflammatory phenotype [47]. EAT is contiguous with the coronary arterial adventitia, allowing inflammatory mediators to diffuse directly into the vessel wall and incite atherogenesis [7] and endothelial dysfunction [48]. There is also a strong association between obesity, EAT inflammation and development of atrial fibrillation [49], myocardial fibrosis, and heart failure [50], which may partly explain the excess cardiac mortality in participants with MetS or NAFLD in our study. The release of adipocytokines from EAT and other visceral fat depots into the general circulation in turn contributes to the systemic inflammatory state associated with obesity [51]. Hence, EAT may act as the local metabolic transducer which mediates the influence of systemic inflammation on the coronary vasculature and myocardium.

### Local influence of EAT on CAC characteristics

We showed regional EAT volume in all three vascular territories to correlate with CAC volume in the respective arteries. Further, there was a positive association between regional EAT attenuation in the LAD and RCA territories and per-vessel calcium density score. It is established that CAC volume is a strong predictor of MACE, and that greater calcium density in plaques has a protective effect [52]. Global EAT volume has been shown to associate with total CAC volume on cardiac CT [53]. Further, we previously reported a graded reduction in global EAT attenuation with an increasing degree of CAC as measured by total Agatston score [13]. The present study is the first to examine the association of location-specific EAT volume and attenuation with CAC characteristics. Our noncontrast CT-based findings suggest that regional EAT may potentially modulate both the volume and density of calcified plaque. This is consistent with recent translational and imaging evidence showing EAT immediately adjacent to the coronary arteries (pericoronary adipose tissue) to play a direct, local role in atherogenesis [7, 54]. The relationship between pericoronary adipose tissue and calcium density should be explored in future studies utilizing CCTA.

### Strengths and limitations

The major strengths of our study include a large sample size, a significant number of outcome events during long-term follow-up, blinded adjudication of endpoints, and an established registry infrastructure. Despite this, the present analysis has several important limitations. First, our clinical data lacks waist circumference assessment, an important element of the definition of central obesity as a MetS component. However, we replaced this criterion with the accepted BMI ≥ 30 kg/m^2^ according to the IDF definition [17]. Pragmatically, evidence suggests that if BMI is greater than 30 kg/m^2^ then waist circumference need not be measured, as over 95% of these individuals will have a waist circumference above the sex- and ethnic-specific threshold values [17]. Second, hepatic attenuation values are affected by other diffuse liver conditions such as iron deposition or hepatitis [8], which can lead to false negatives or false positives, respectively, for steatosis. Third, our DL technique for EAT quantification is novel and may potentially introduce measurement bias. Although expert manual EAT annotations were not performed in the EISNER cohort, our DL algorithm did not exhibit significant bias when validated against expert readers in a large multicenter study (0.53 cm^3^, p = 0.13) [10]. Finally, the EISNER trial was subject to sample bias as it comprised mostly Caucasian, highly educated, and fairly affluent volunteers. All participants were asymptomatic with no known CAD. Hence, our results may not be generalizable to different demographics or symptomatic patients with CAD.

## Conclusion

MetS, NAFLD, and AI-based EAT measures predict long-term risk of MACE in asymptomatic individuals. Imaging biomarkers of cardiometabolic disease have the potential for integration into routine reporting of CAC scoring CT to enhance cardiovascular risk stratification.

## Supplementary information

**Additional file 1: Table S1.** Relationship of EAT measures with individual components of the MetS. **Table S2.** Relationship of EAT measures with presence of MetS in multivariable logistic regression. **Table S3.** Relationship of EAT measures with NAFLD in multivariable logistic regression. **Table S4.** Levels of serum biomarkers according to the presence vs absence of MetS or NAFLD. **Figure S1.** Case example of artificial intelligence-based regional EAT quantification. **Figure S2.** Case example of liver and spleen attenuation measurement on noncontrast CT.

## Data Availability

The datasets used and/or analyzed during the current study are available from the corresponding author on reasonable request.

## Abbreviations

ASVCD: Atherosclerotic cardiovascular disease
BMI: Body mass index
CAC: Coronary artery calcium
CAD: Coronary artery disease
CT: Computed tomography
DL: Deep learning
EAT: Epicardial adipose tissue
HU: Hounsfield units
MACE: Major adverse cardiovascular events
MetS: Metabolic syndrome
NAFLD: Non-alcoholic fatty liver disease
