# Supplementary material for: Metabolic syndrome, fatty liver, and artificial intelligence-based epicardial adipose tissue measures predict long-term risk of cardiac events: a prospective study
Source: Cardiovasc Diabetol. 2021 Jan 29;20:27. doi: 10.1186/s12933-021-01220-x (PMC7847161; doi:10.1186/s12933-021-01220-x)
Supplement: Supplementary file 1 — Additional file 1: Table S1. Relationship of EAT measures with individual components of the MetS. Table S2. Relationship of EAT measures with presence of MetS in multivariable logistic regression. Table S3. Relationship of EAT measures with NAFLD in multivariable logistic regression. Table S4. Levels of serum biomarkers according to the presence vs absence of MetS or NAFLD. Figure S1. Case example of artificial intelligence-based regional EAT quantification. Figure S2. Case example of liver and spleen attenuation measurement on noncontrast CT. [file 12933_2021_1220_MOESM1_ESM.docx]

**Table S1.** Relationship of EAT measures with individual components of the MetS

| **MetS components** | **Correlation**  **(EAT volume)** | ***P* value** | **Correlation**  **(EAT attenuation)** | ***P* value** |
| --- | --- | --- | --- | --- |
| Body mass index | 0.54 | **<0.001** | -0.35 | **<0.001** |
| Serum triglycerides | 0.32 | **<0.001** | -0.32 | **<0.001** |
| HDL cholesterol | -0.35 | **<0.001** | 0.28 | **<0.001** |
| Systolic blood pressure | 0.20 | **<0.001** | -0.14 | **<0.001** |
| Diastolic blood pressure | 0.24 | **<0.001** | -0.12 | **<0.001** |
| Fasting glucose | 0.19 | **<0.001** | -0.15 | **<0.001** |

Abbreviations: EAT, epicardial adipose tissue; HDL, high-density lipoprotein; MetS, metabolic syndrome.

**Table S2.** Relationship of EAT measures with presence of MetS in multivariable logistic regression

|  | | | |
| --- | --- | --- | --- |
|  | **OR** | **95% CI** | ***P* value** |
| **Model 1 - EAT volume** | | | |
| EAT volume, cm^3^* | 7.83 | 5.90-10.39 | **<0.001** |
| Age, years | 0.98 | 0.96-0.99 | **0.03** |
| Male sex | 0.60 | 0.44-0.81 | **0.001** |
| Smoking | 0.92 | 0.53-1.60 | 0.77 |
| LDL cholesterol, mg/dL | 1.00 | 0.99-1.00 | 0.11 |
| CAC score* | 1.01 | 0.97-1.06 | 0.68 |
|  |  |  |  |
| **Model 2 - EAT attenuation** | | | |
| EAT attenuation, HU | 0.85 | 0.83-0.88 | **<0.001** |
| Age, years | 0.99 | 0.98-1.01 | 0.46 |
| Male sex | 0.86 | 0.65-1.14 | 0.29 |
| Smoking | 1.14 | 0.67-1.95 | 0.63 |
| LDL cholesterol, mg/dL | 1.00 | 0.99-1.00 | 0.13 |
| CAC score | 1.03 | 0.99-1.08 | 0.14 |

*Odds ratios are per 2-fold increase/doubling of EAT volume (cm^3^) and CAC score.

Abbreviations: CAC, coronary artery calcium; CAD, coronary artery disease; EAT, epicardial adipose tissue; HU, Hounsfield units; LDL, low-density lipoprotein.

**Table S3.** Relationship of EAT measures with NAFLD in multivariable logistic regression

|  | | | |
| --- | --- | --- | --- |
|  | **OR** | **95% CI** | ***P* value** |
| **Model 1 - EAT volume** | | | |
| EAT volume, cm^3^* | 2.34 | 1.70-3.22 | **<0.001** |
| MetS | 3.72 | 2.53-5.51 | **<0.001** |
| Age, years | 0.99 | 0.97-1.01 | 0.08 |
| Male sex | 1.03 | 0.71-1.51 | 0.88 |
| Smoking | 1.32 | 0.71-2.44 | 0.38 |
| LDL cholesterol, mg/dL | 1.00 | 1.00-1.01 | 0.07 |
| CAC score* | 1.04 | 0.99-1.10 | 0.15 |
|  |  |  |  |
| **Model 2 - EAT attenuation** | | | |
| EAT attenuation, HU | 0.89 | 0.86-0.92 | **<0.001** |
| MetS | 4.26 | 2.94-6.18 | **<0.001** |
| Age, years | 0.98 | 0.96-1.00 | 0.05 |
| Male sex | 1.12 | 0.77-1.63 | 0.57 |
| Smoking | 1.48 | 0.80-2.76 | 0.21 |
| LDL cholesterol, mg/dL | 1.00 | 0.99-1.01 | 0.10 |
| CAC score | 1.04 | 0.99-1.10 | 0.14 |

*Odds ratios are per 2-fold increase/doubling of EAT volume (cm^3^) and CAC score.

Abbreviations: CAC, coronary artery calcium; CAD, coronary artery disease; EAT, epicardial adipose tissue; HU, Hounsfield units; LDL, low-density lipoprotein; NAFLD, non-alcoholic fatty liver disease.

**Table S4.** Regional EAT measures and per-vessel CAC characteristics according to vascular territory

|  | **LAD** | **LCx** | **RCA** |
| --- | --- | --- | --- |
| EAT measures |  |  |  |
| EAT volume, cm^3^ | 27.1 (19.5-40.4) | 21.2 (16.3-31.5) | 38.4 (28.4-52.7) |
| EAT attenuation, HU | -76.9±5.4 | -75.4±5.3 | -74.2±5.2 |
|  |  |  |  |
| CAC characteristics* | (n=876) | (n=414) | (n=475) |
| Agatston score | 50.9 (14.4-142.3) | 26.4 (6.5-80.8) | 39.3 (9.8-142.5) |
| Volume, cm^3^ | 39.3 (11.5-107.7) | 22.0 (6.8-61.7) | 36.4 (9.3-121.1) |
| Area, cm^2^ | 14.5 (4.3-40.8) | 8.2 (2.6-24.2) | 13.5 (3.6-43.0) |
| Calcium density score | 3.6 (2.9-4.0) | 3.1 (2.5-3.6) | 3.1 (2.4-3.6) |

*Results for subjects with CAC present in the respective vessel.

Abbreviations: CAC: coronary artery calcium; EAT: epicardial adipose tissue; HU: Hounsfield.

**Table S5.** Levels of serum biomarkers according to the presence versus absence of MetS or NAFLD

| **Serum biomarkers** | **MetS**  (n=145) | **No MetS**  (n=924) | ***P* value** | **NAFLD**  (n=131) | **No NAFLD**  (n=938) | ***P* value** |
| --- | --- | --- | --- | --- | --- | --- |
| hs-CRP, μg/mL | 4.3 (2.3-10.2) | 2.0 (0-4.6) | **<0.001** | 4.3 (2.0-10.7) | 2.2 (0-5.1) | **<0.001** |
| IL-6, pg/mL | 10.2 (4.0-16.4) | 5.8 (1.8-14) | **<0.001** | 9.7 (3.4-16.1) | 6.0 (1.9-14.8) | **0.02** |
| PAI-1, ng/mL | 22.0 (15.3-30.5) | 12.2 (6.8-19.4) | **<0.001** | 24.8 (15.7-33.7) | 12.7 (7.2-20.2) | **<0.001** |
| D-dimer, ng/mL | 168.4 (84.5-350.9) | 119.9 (61.1-226.6) | **<0.001** | 174.8 (103.2-320.0) | 122.3 (62.8-241.3) | **<0.001** |
| MPO, pmol/L | 286.9 (230.5-408.9) | 241.4 (181.3-340.2) | **<0.001** | 285.2 (230.6-416.2) | 245.0 (183.5-346.8) | **<0.001** |
| ESAM, ng/mL | 26.6 (21.6-38.1) | 24.0 (18.9-30.9) | **<0.001** | 27.2 (21.6-37.3) | 23.9 (19.0-31.2) | **0.004** |
| LTBR, ng/mL | 0.90 (0.70-1.30) | 0.80 (0.60-1.10) | **<0.001** | 0.90 (0.60-1.20) | 0.80 (0.60-1.10) | **0.02** |
| Adiponectin, ng/mL | 3685 (1999-6446) | 5590 (3080-9976) | **<0.001** | 3005 (1935-5189) | 5497 (3021-9986) | **<0.001** |

Values are expressed as median (interquartile range, 25^th^-75^th^).

Abbreviations: ESAM, endothelial cell-selective adhesion molecule; hs-CRP, high-sensitivity C-reactive protein; IL-6, interleukin-6;
LTBR, lymphotoxin β receptor; MetS, metabolic syndrome; MPO, myeloperoxidase; PAI-1, plasminogen activator inhibitor 1.

**Figure S1. Case example of artificial intelligence-based regional EAT quantification**
Eight EAT segments based from the center of the heart in the axial view were automatically generated by the deep learning software. EAT segments were assigned to the best suitable coronary artery according to myocardial vascular territories. Regional EAT volume corresponding to each major artery was calculated from the vessel-assigned EAT segments; EAT attenuation was the mean attenuation of these regions.

Abbreviations: EAT: epicardial adipose tissue; LAD: left anterior descending artery; LCx: left circumflex artery; RCA: right coronary artery

**
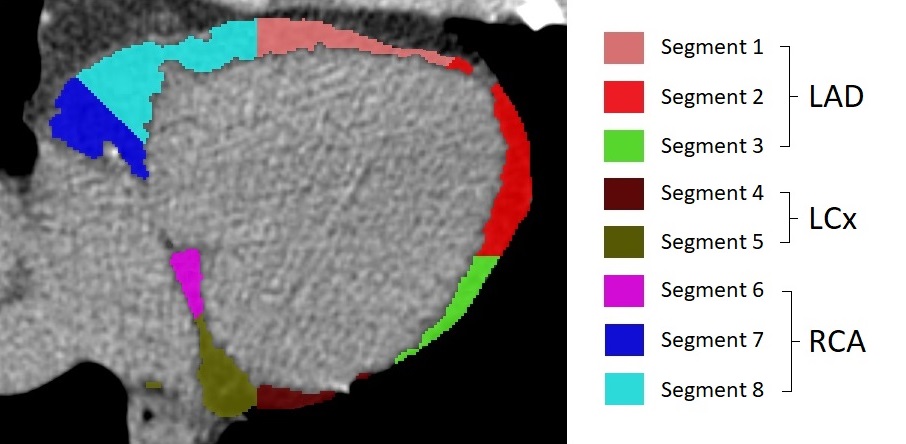
**

**Figure S2. Case example of liver and spleen attenuation measurement on noncontrast CT**
In the same axial slice, two ROI were placed in the right liver lobe anteroposteriorly, one ROI in the left liver lobe, and one ROI in the spleen. Liver-to-spleen ratio was calculated by taking the mean HU measurement of both right liver lobe ROIs and dividing it by the splenic HU. Average liver attenuation was the mean HU measurement of right and left lobe ROIs. NAFLD was defined as liver-to-spleen ratio <1.0 and/or average liver attenuation <40 HU.

Abbreviations: CT, computed tomography; HU, Hounsfield units; NAFLD, non-alcoholic fatty liver disease; ROI, regions of interest.

**
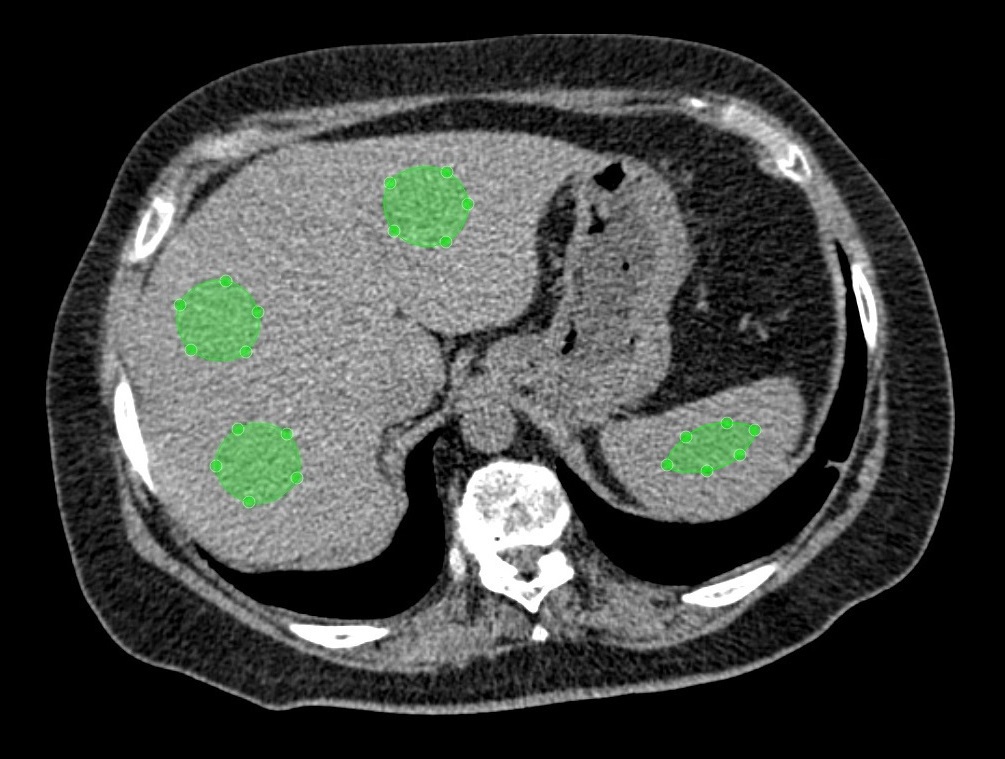
**
